# The Impacts of Climate and Social Changes on Cloudberry (Bakeapple) Picking: a Case Study from Southeastern Labrador

**DOI:** 10.1007/s10745-018-0038-3

**Published:** 2018-11-20

**Authors:** Darya Anderson, James D. Ford, Robert G. Way

**Affiliations:** 1 McGill University, Montreal, QC Canada; 2 Priestley International Centre for Climate, University of Leeds, Leeds, UK; 3 Department of Geography and Planning, Queen’s University, Kingston, ON K7L3N6 Canada; 4 Labrador Institute, Memorial University of Newfoundland, Happy Valley-Goose Bay, NL A0P1E0 Canada

**Keywords:** Climate change, Vulnerability, Cloudberry (bakeapple) picking, Indigenous peoples, Subarctic, Southeastern Labrador, Canada

## Abstract

The traditional subsistence activities of Indigenous communities in Canada’s subarctic are being affected by the impacts of climate change, compounding the effects of social, economic and political changes. Most research has focused on hunting and fishing activities, overlooking berry picking as an important socio-cultural activity and contributor to the diversity of food systems. We examined the vulnerability of cloudberry (referred to as ‘bakeapple’ consistent with local terminology) picking to environmental changes in the community of Cartwright, Labrador using semi-structured interviews (*n* = 18), field surveys, and satellite imagery. We identified the components of vulnerability including: the environmental changes affecting the abundance, quality, and ripening time of bakeapples (i.e., exposure), the characteristics of the community that affect how these changes have local impacts (i.e., sensitivity), and the ways in which the community is responding to environmental changes (i.e., adaptive capacity). Our results confirm that environmental changes related to permafrost, vegetation, and water have occurred at the bakeapple picking grounds with observed impacts on bakeapples. It is becoming increasingly difficult for bakeapple pickers to respond to variable growth as in the past because of changes in summer settlement patterns that place families farther from their bakeapple patches. We conclude that harvesters in Cartwright have high adaptive capacity to respond to environmental changes due to their knowledge of their bakeapple patches, and at present, socioeconomic changes have had a greater impact than environmental changes on their harvesting capacity.

## Introduction

Global temperatures are increasing at unprecedented rates with increases amplified in the Arctic (IPCC 2013; AMAP 2017). According to Environment Canada, air temperatures have increased by 1.7 °C and by 3.1 °C in the western Canadian Arctic. Indigenous communities in the Arctic and Subarctic, in particular, are highly sensitive to climate changes (Serreze and Barry 2011) because of their close relationship with the land for livelihoods and associated food systems, colonial legacies, and concurrent socioeconomic changes (Anisimov *et al.* 2007; Ford *et al.* 2015).

Scholarship examining the human dimensions of climate change in the Arctic and Subarctic, particularly in Canada and with a focus on Indigenous peoples is well established (Ford *et al.* 2018). Much of this work describes hunting and fishing livelihoods and associated food systems, but comparable work on berry picking is largely absent (Ford *et al.* 2012b; Bunce *et al.* 2016). There are also geographic disparities in research, with most scholarship focusing on communities and the region above 60°N (Ford *et al.* 2012a) rather than the Subarctic (Downing and Cuerrier 2011; Cuerrier *et al.* 2015).

Wild berry picking is an important part of the food subsistence for Indigenous communities in southern Labrador, for both nutritional and cultural reasons (Fitzhugh 1999). Individuals consider wild berries, including bakeapples, to be healthier than store bought foods and berry picking traditions have been passed down for generations. There is a two-to-three week window, typically in August, when families go bakeapple picking. Bakeapples often grow in peatlands on or around palsa features (a peat mound with a permafrost core) and many factors including precipitation, temperature, soil moisture, and the surrounding vegetation impact their growth (Korpelainen 1994; Kellogg *et al.* 2010; Seppälä 2011). Models predict that most peatland permafrost in southeastern Labrador will thaw by the end of the twenty-first century (Way *et al.* 2018). Thawing of peatland permafrost can cause ground subsidence, hydrological changes, thermokarst development, and fragmentation of the palsa dependent vegetation—forbs, shrubs, lichens— and a transition to wet graminoids and *Sphagnum moss* spp. (Camill 1999; Camill *et al.* 2001; Christensen *et al.* 2004; Johansson *et al.* 2006). While the importance of palsas for bakeapple growth is unclear, previous research suggests impacts of climate related changes on the ripening time, quality, and abundance of berries (Cuerrier *et al.* 2015).

We identify and characterize the vulnerability of bakeapple (*Rubus chamaemorus* and Appik in Inuttitut) picking to climate change, combining qualitative and quantitative methods including interviews, field surveys, and satellite imagery analysis. Focusing on the community of Cartwright, Labrador, we utilize a ‘contextual approach’ to vulnerability assessment, focusing on both climatic and non-climatic factors affecting berry picking.

## Methodology

### Conceptual Approach

We structured this research using a vulnerability approach. Vulnerability can be defined as the “susceptibility to be harmed” (Adger 2006:269) with vulnerability research seeking to identify and characterize who is vulnerable, to what stresses, and why (Ford and Smit 2004). Contextual vulnerability considers the political, biophysical, social, and economic factors that interact to shape vulnerability and defines vulnerability as a function of exposure, sensitivity, and adaptive capacity (O’Brien *et al.* 2007; Wang *et al.* 2014; Archer *et al.* 2017); exposure captures the environmental changes affecting the ripening time, abundance and quality of berries; sensitivity reflects the livelihoods and other social, economic, and political characteristics of the community, and shapes the impacts of exposure, e.g., if there is a landslide caused by permafrost degradation (exposure), the effect on a community will be dependent on components of sensitivity such as the location of settlements, infrastructure, land use, and technology (Smit and Wandel 2006); adaptive capacity reflects the potential ability of the community to plan for, address, or cope with exposure (Ford and Smit 2004; Smit and Wandel 2006; O’Brien *et al.* 2007; Bennett *et al.* 2016), e.g., berry pickers may bring extra gasoline anticipating the need to visit multiple bakeapple picking grounds by boat. The response to these drivers of vulnerability in turn shapes and changes the initial conditions of vulnerability, and as a result, vulnerability is dynamic (Smit and Wandel 2006; Ford *et al.* 2013; Archer *et al.* 2017; Fawcett *et al.* 2017). Case studies are particularly important for identfiying the various factors affecting vulnerability and understanding how they interact together in particular places (Ford *et al.* 2010).

### Case Study Location

**Fig. 1:**
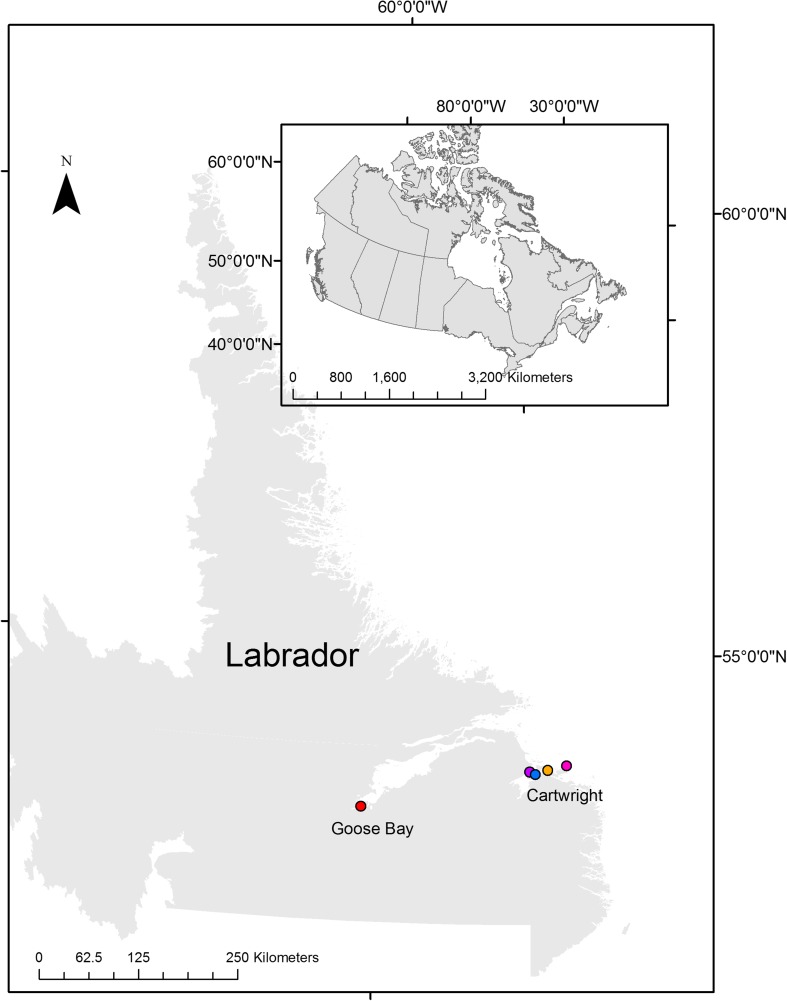
A map of Canada and a zoomed in version of Labrador with Cartwright and Happy-Valley Goose Bay labeled. The purple (Main Tickle Point), orange (Hare Harbor), pink (Grady), and blue (The Big Marsh) mark field survey sites. Data source: Statistics Canada (https://www12.statcan.gc.ca/census-recensement/2011/geo/bound-limit/bound-limit-ieng.cfm?year=18) for Cartographic Boundary File of Canada and Elections Newfoundland and Labrador (2015) for Labrador shape file

Cartwright is located in southern Labrador on Sandwich Bay on the Atlantic coast (Fig. 1) in the mid Boreal Forest ecoregion (Roberts *et al.* 2006). According to Environment and Natural Resources Canada (1981–2010 climate normal), Cartwright had an average annual temperature of 0 °C, experienced average annual rainfall of 616.8 mm and average annual snowfall of 462 cm. The permafrost is classified as isolated patches and is limited to palsa features in peatlands, which are between 0.3–1.3 m high and covered by lichens and exposed peat (Way *et al.* 2018). In the surrounding wetter depressions with no permafrost shrubs and mosses predominate. Three important salmon spawning rivers feed into Sandwich Bay. The regional flora and fauna are ecologically diverse including seals, whale, black bear, polar bear, caribou, wolf, fox, small mammals, waterfowl, marine and freshwater fish, blueberries, crowberries, bakeapples, lichen, white and black spruce, Eastern larch, and birch (Roberts *et al.* 2006).

According to the Canadian 2016 census, Cartwright has a population just under 500 individuals, the majority with Indigenous ancestry and recognized as the people of NunatuKavut. Due to the Government of Newfoundland and Labrador’s centralization program in the 1960s, Cartwright was provided with a school, a grocery store, postal service, telephone lines, and a road (Lethbridge and Chesley 2007), causing many families in nearby communities without these services to move to Cartwright. Presently, many individuals are employed at the Labrador Fishermen’s Union Shrimp Company Limited where crab is processed from the offshore crab fishery. Historically, there was a cod fishery but stocks declined noticeably in the second half of the twentieth century and the Canadian government placed an official moratorium on the commercial cod fishery throughout Southern Labrador in 1992. The inland commercial salmon fishery closed a few years later.

### Data Collection and Analysis

Mixed methods — field surveys, satellite imagery analysis, interviews —were used to characterize the environmental changes affecting the ripening time, abundance, and quality of bakeapples (exposure). Interviews were also used to characterize sensitivity and adaptive capacity, drawing upon the traditional ecological knolwedge (TEK) of informants. Traditional knowledge (TK) is the “cumulative body of knowledge, practice, and values acquired through experience and observations on the land or from spiritual teachings and handed down from generation to generation” (Pearce *et al.* 2015: 235). TEK is a subset of TK that specifically encompasses knowledge about the environment and human’s relationship with it (Riedlinger and Berkes 2001). TEK is an integral part of the ability of Indigenous communities to safely and effectively live off the land, continues to be a component of adaptive capacity to climate change, and is increasingly valued by researchers seeking to understand how climate change interacts with society (Huntington 2011; Pearce *et al.* 2015).

### Interviews

We conducted semi-structured interviews with 18 individuals (Longhurst 2003) with an interview guide (Valentine 1997) *—* structured by the components of vulnerability (Table 1). The interviews lasted between 30 min to an hour. A purposive sampling strategy (Baxter and Eyles 1997) was used to select participants with rich knowledge and experience of bakeapple picking and was implemented with the help of a respected and active community member and then via a snowballing strategy, in which interviewees suggested other potential participants. Interviews were conducted until a saturation of relevant information was reached.

**Table 1:** Themes with corresponding component of vulnerability and sample questions from interview guide

Themes	Example questions
Duration of time an individual has picked for	About how many years have you been bakeapple picking?
Observations of environmental changes at the bakeapple picking grounds (exposure)	Have you noticed changes in the plant species where you pick bakeapples?
	Have you noticed a change in the amount of surface water in the landscape?
	How much variability is there from year to year in the abundance and quality of bakeapples?
	What things have affected how good the bakeapples are in a particular year?
	Has the timing of when pick bakeapples changed from your youth?
Observations of social change related to bakeapple picking (sensitivity)	What importance does bakeapple picking have to you currently as an adult and previously as a child?
	What does an average day of bakeapple picking look like now and as a child?
Observations of economic change related to bakeapple picking (sensitivity)	Do you sell the bakeapples that you pick?
	Have job opportunities impacted your ability to go bakeapple picking?
Technology advances and impact on bakeapple picking (sensitivity)	How do you get to the spots that you bakeapples pick now and is this different from your youth?
The main barriers and reasons for bakeapple picking (adaptive capacity)	What are your greatest barriers to go bakeapple picking? How do you deal with these barrier(s)?
	What are the main reasons that you go bakeapple picking?

Interviews were coded to identify the key components of the vulnerability of bakeapple picking to changes in the physical and social landscape. The 14 recorded interviews were transcribed (Bedford and Burgess 2001). For the four unrecorded interviews, annotations and memos related to the method or context were made on the transcriptions and on the interview notes (Dunn 2000; Cope 2003; Corbin and Strauss 2014). Based on the emerging patterns, initial codes were created, and based on how they connected to the components of vulnerability secondary codes were generated (Cope 2010). Finally, we identified the emerging key themes based on the secondary codes and our knowledge of the literature (Saldaña 2013).

### Field Surveys

The relevé method was used to determine the importance of permafrost conditions on the presence of bakeapples to describe the exposure. A relevé is, at minimum, a list of present species within the plot (Causton 1988). Plots spanned four permafrost-associated peatlands (Fig. 1) and surveying occurred over four consecutive days (August 7–10, 2017). The plots were 1m^2^, based on recommendations for lichen and moss communities (Ellenberg and Mueller-Dombois 1974). Plots were stratified between palsa sites with an active layer overlying permafrost, and sites with no detectable permafrost within 120 cm of the peat surface. In total, 31 plots were randomly selected on both palsa and no permafrost sites by throwing the quadrat in areas of the peatland with palsa features. Seven palsa and seven no permafrost plots were selected at Hare Harbor; eight palsa and eight no permafrost plots at Grady; five palsa and five no permafrost plots at Main Tickle Point; 11 palsa and 11 no permafrost plots at The Big Marsh in Cartwright. Per plot, species frequency was measured with the Daubenmire scale (Ellenberg and Mueller-Dombois 1974; Thomas *et al.* 2003). Elevation was measured with a hand-held Garmin GPS with a resolution of less than 10 m, and active layer depth (ALD) was measured with a 120 cm permafrost probe.

R [1.0.143] was used to analyze the field data. To determine if bakeapples are significantly different across palsas and no permafrost sites, a Fisher’s exact chi squared test was used with the *stats* package (Causton 1988). With the Vegan package, non-metric multidimensional scaling (NMDS), using the Bray-Curtis dissimilarity index, was performed to determine if distinct vegetation communities are associated with the palsa and no permafrost plots (Šmilauer and Lepš 2014); ANOSIM analysis was done to test whether the dissimilarity between the vegetation of the palsa and no permafrost plots is significantly greater than the dissimilarity within each group; SIMPER analysis was done to determine which species are contributing most to dissimilarity between groups (Clarke 2006). Spearman rank correlation coefficients were calculated with the stats package to determine if elevation and/or ALD are significantly correlated with the NMDS coordination axes; elevation and ALD were then fitted via regression to the ordination axes using the Vegan package (Causton 1988).

### Remote Sensing

Using ENVI 5.4 software, area changes for palsas, vegetation, and surface water were assessed at The Big Marsh *—*all of which contributed to the characterization of the exposure. This specific peatland was evaluated because it was frequented by community members for berry picking, palsas were present in the bog, and it overlapped with readily accessible satellite imagery. Courtesy of the DigitalGlobe Foundation, a 2004 Quickbird image and a 2016 World View 2 image were acquired — both of which have fine enough spatial resolution to detect palsas. Pre-proccessing of images included radiometric and atmospheric correction with the FLAASH algorithm via tools available through ENVI 5.4. The 2004 and 2016 images were co-registered following the approach of Andresen and Lougheed (2015). For this study, there were a limited number of identifiable tie points due to a coarser resolution in the 2004 image and little built infrastructure surrounding the peatland, so 11 tie points were used with a final RMSE of 1.90 m.

For palsas, the manual target detection approach used by Beck *et al.* (2015) and Bouchard *et al.* (2014) was applied. Additionally, following the approach of Sannel and Kuhry (2011), we used the panchromatic bands with the aid of the multispectral bands to identify palsas. Automatic target detection, with the matched Filtering (MF) algorithm, was used to identify any green vegetation and surface water in the 2004 and 2016 images. To assess the target detection, the separability between vegetation, water, and the unclassified areas was computed with Jeffries-Matusita separability measures. All combinations of features had Jeffries-Matusita separability measures greater than 1 indicating good spectral separability (Richards and Jia 2006). Following Richards and Jia (2006), we calculated the following classification accuracy metrics: overall accuracy, producer accuracy, and user accuracy. The user accuracy, which is the chance that a pixel labeled vegetation or water is actually vegetation or water, was 81.82 and 87.50% for the surface water class in 2004 and 2016 respectively, and 100% for the vegetation class in both 2004 and 2016. The producer accuracy and the overall accuracy were lower for both classes meaning that not all vegetation or water reference pixels were classified as vegetation or surface water pixels during the automatic target detection.

## Results

We first describe the activity of bakeapple picking and the ecological conditions at the picking grounds before characterizing the components of vulnerability drawing upon interviews and quantitative data. Note that interviewees agreed to the use of their quotes and some requested that their names be cited.

### Bakeapple Picking

In 17 of the 18 interviews, participants said that bakeapple picking is a family activity.

“We did it with our two children from the time we had them till they left and then the grandkids came along and we are still doing it. 46 years of doing that.” Judy Pardy

Half of the interviewees explained that in the past if the men were fishing only the women and children would bakeapple pick.

Every family has their favorite spots to go bakeapple picking, some of which go back generations and other new ones are discovered by word of mouth. Often, a family keeps the exact location of their favorite picking grounds a secret to protect their supply. There is consensus among all interviewees that the best bakeapple picking areas are on the outer islands, and during the harvest, usually in August, families travel by boat to their summer homes or islands nearby.

Bakeapples are used for special dishes like pies, cheesecakes, and muffins during the holidays. For some individuals, bakeapples are a part of their daily diet, although this was more common in the past when local grocery stores did not sell fruit during the winter.

“Well, my father, he was 97 when he died, he had bakeapples every day. Every day for breakfast he had bakeapples.” Dwane Burdett

Families will generally go for day trips or a holiday to the bakeapple grounds from the end of July through September, depending on when the bakeapples ripen. Largely, interviewees consider this variability to be normal.

“Timing changes almost every year. You might be able to pick bakeapples in the middle of July and most times it is not until the first week in August and then from that on the bakeapples ripen.” Leslie Hamel

Almost half of the participants describe how the temperatures in both the spring (April and May) and the summer (June, July, and August) determine the ripening time. With a shorter spring and earlier summer the bakeapples will ripen sooner. Also, about a third of participants explain that the time when bakeapples ripen depends on location, and can be as late as early September if the berries are in a sheltered spot where they receive less sun or on an island farther from the mainland.

The quantity that a family picks depends on their uses of the bakeapples. Generally, if a family is picking solely for its own use, they will pick between five to 15 gal per season. If the family is picking with the intention of selling, they might pick anywhere from 20 to 50 gal depending on the abundance of bakeapples in a given year. Participants expect the growth of bakeapples to be variable from year to year and largely attribute this to weather.

“Some years there’s none nowhere! I’ve been up there and sometimes you can literally see that the land has an orange glow to it. Like an orange… a big orange carpet.” Dwane Burdett

About a third of participants emphasized that the weather during the spring and summer of the year is a key factor in the abundance of that season. Interviewees explained that in the spring if there are strong winds (between 40 to 60 km/h) or a downpour of rain then the bakeapple blossom can be destroyed because it is particularly sensitive until the shuck has closed in. Interviewees also explain that the weather during the summer is very important. Extreme warm temperatures (above 25° Celsius) can destroy the fruit.

“If it’s too hot they’ll kind of burn up… and if it’s cold and damp for a while they just won’t grow like they should. You’ll get a few but they’ll be small or spaced apart.” Tracy Martin

In addition to the importance of weather, ecological constraints on bakeapple growth were identified and characterized by interviewees and through field surveys. Interviewees described the optimal ecological conditions for bakeapple growth. Almost all explained that bakeapples are found in the boggy but not overly wet areas.

“A lot of the places you pick bakeapples are pretty much wet spots anyways. Not right wet as such but damp… the best areas.” Leslie Hamel

Additionally, more than half (*n* = 11) explained that bakeapples will likely be larger and more abundant in spots sheltered by the (micro)topography of the landscape and/or surrounding plants.

**Fig. 2:**
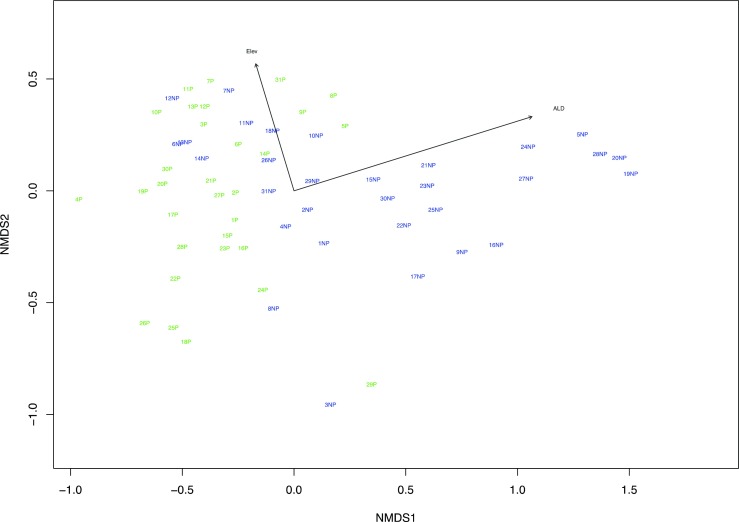
NMDS showing the relative position of the vegetation plots in a two-dimensional space via comparison of the species surface cover per plot. Palsa (P) plots are in green and no permafrost (NP) plots are in blue. Proximity of plots implies less species composition dissimilarity. Environmental vectors (Elev = elevation, ALD = active layer depth) are overlaid on the NMDS. The direction of the vector indicates whether the correlation is positive or negative and the magnitude of the vector is proportional to the strength of the correlation

We conducted field surveys to determine whether the presence or absence of permafrost in palsa bogs constrained the presence or absence of bakeapples. A chi-squared test showed that the presence/absence of bakeapples is not significantly different between palsa and no permafrost plots. However, ANOSIM analysis of a NMDS plot of the vegetation data revealed that the vegetation types between palsa and no permafrost plots are significantly different with an ANOSIM test statistics equal to 0.226 and a significance value of 0.001 (Fig. 2). Additionally, SIMPER analysis showed that *sphagnum moss* spp*., sedge* spp*. Empetrum nigrum, Lichen* spp*.,* bakeapple (*Rubus chamaemorus)*, *Vaccinium ulginosum, and Ledum groelandiculum* contributed to 70% of the dissimilarity between palsa and no permafrost plots. Spearman rank correlation coefficients revealed that ALD was significantly correlated with the first ordination axes and elevation was significantly correlated with the second ordination axes and therefore, both ALD and elevation explain differences in the vegetation types between the palsa and no permafrost plots (Fig. 2). While permafrost presence in palsa bogs does not significantly impact the presence/absence of bakeapples*,* it does have a significant impact on vegetation species and the ecosystem as a whole (Photograph 1).

**Photograph 1:**
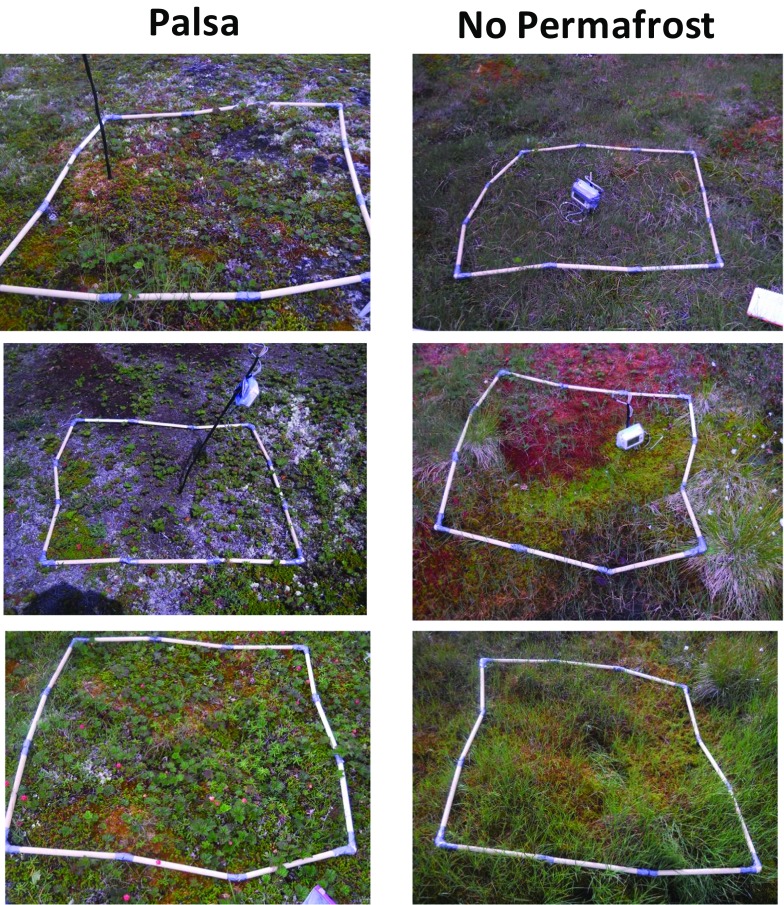
Examples of palsa (left) and no permafrost (right) vegetation types

### Exposure

Participants discussed ecological changes that are beyond past experiences and related to the growth of bakeapples, noting that knowledge on change varied by individual for some observations. While this reflects lack of consensus in some observations (e.g., for weather, hydrological conditions, abundance of bakeapples), it also reflects differential engagement of individuals with bakeapple picking and different locations regularly visited.

**Fig. 3:**
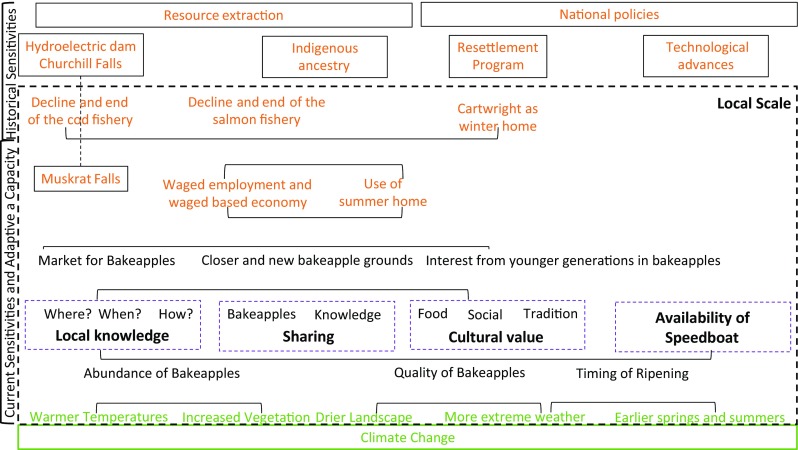
A diagram of the vulnerability of bakeapple picking to physical and social changes. The exposures are in green; the adaptive capacities are in purple; the sensitivities are in orange. The local scale is marked with a dotted black square. The current and historical components are indicated on the left of the figure. Arrows show the interactions between different components

A few interviewees consider the recent variability in weather, including wind and temperature, to be beyond normal (Fig. 3).

“The berry picking for the bakeapples has changed because the weather has changed. The weather has changed, because it is more windy. A lot of wind. And more stormy. A lot of storms…. Extreme weather conditions are changing the bakeapples.” Rosetta Howell

While these participants consider spring temperatures to be warmer in the last decade they also noted that the 2017 spring was an exception, being colder than usual.

Almost half the participants observed that bakeapples have been ripening earlier than normal in recent years (Fig. 3), and explained that this is because of changes in the timing of the seasons and earlier warmer temperatures.

“The springs are coming earlier each year and because of that your blossoms are coming out earlier and they are ripening earlier because of that.” Dave Hamel

A few participants independently attributed changes in the growth of bakeapples to climate change.

Ten participants noted changes in the landscape at the bakeappple picking grounds, and many observed increases in larger shrubs surrounding bakeapple grounds and felt this vegetation is competing with the bakeapples (Fig. 3).

“Some of the other shrubs seem to be overtaking some of the… places where bakeapples grow.” Dave Hamel

**Fig. 4:**
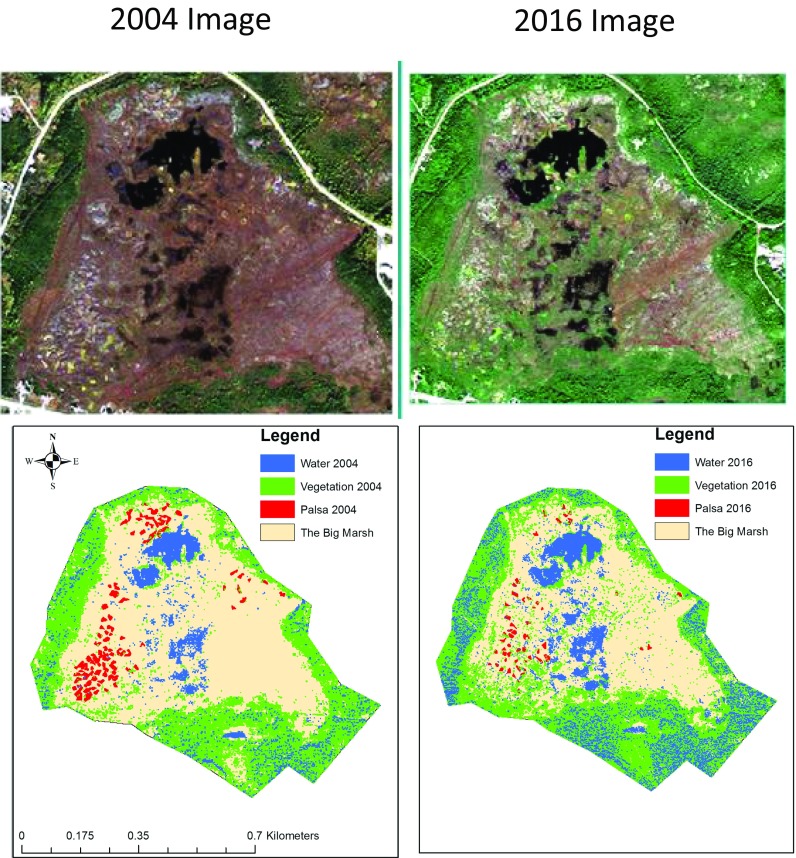
The Big Marsh in 2004 (left) and 2016 (right). Palsa, vegetation, and surface water delineated in 2004 and 2016. The unclassified areas are bare ground. Satellite Imagery courtesy of the DigitalGlobe Foundation

Analysis of satellite images from 2004 to 2016 indicates an increase in total green vegetation in The Big Marsh by 21.5% (Fig. 4).

Some participants, in response to a question about changing water conditions at bakeapple picking grounds reported that the landscape is drier (Fig. 3) as could be observed through small bodies of water in the landscape, ponds and rivers, or by the kinds of shoes needed to go picking. Several participants described how a small pond near a popular bakeapple picking ground completely dried up one year after large cracks formed in the landscape. Satellite imagery showed that the surface water increased at The Big Marsh from 2004 to 2016 by 66.4%, consistent with the development of thermokarst (Fig. 4). It is possible that this discrepancy is because bakeapple pickers would tend to avoid the grounds that are becoming wetter because they became less accessible.

A third of participants observed that the bakeapple fruits are changing and that they grow differently, but did not know why (Fig. 3). A few observed that the way the bakeapples are ripening is more fragmented in space and time.

“But I know that the bakeapples don’t grow anymore like they used to years ago. Years ago, when they ripe, you go out into a spot of bakeapples and you just pick, pick, pick. But now when you go you pick a few that’s ripe and then you go back a bit later and pick a few more when they are ripe. Why? I don’t know. But I do know there is a lot of vegetation growing up that wasn’t in some places anyhow.” Donnie Howell

Fragmentation of bakeapple patches may be related to changes in palsas., palsas are estimated to have decreased in area from 2004 to 2016 by 64.5% at The Big Marsh (Fig. 4). From relevé analysis, it is evident the vegetation is different on palsa features compared to the surrounding areas with no permafrost, and is significantly correlated with changes in elevation and ALD. The decrease in palsa features in The Big Marsh has likely contributed to i) changes in vegetation communities resembling the differences, at present, between the palsa and no permafrost sites, and ii) fragmentation of the previous permafrost palsa vegetation communities, including bakeapple patches.

In addition to these biophysical stressors, socioeconomic changes are impacting where and when families pick bakeapples.

### Sensitivity

Sensitivity to the changes in exposure documented needs to be understood in regard to long-term changes in human-environment interaction. Before the 1960s, families moved between summer homes, conveniently located for fishing and bakeapple picking, and winter homes for trapping and hunting. Resettlement to Cartwright in the 1960s moved community members farther from their summer homes and picking grounds making it harder to cope with variability in the growth of bakeapples.

However, before the moratorium on cod and salmon fishing in the 1990s, many families went to their summer homes out on the islands around June and returned to Cartwright around September. The salmon fishery in July ended just before the bakeapple harvest in August, when the cod fishery also started. After the moratorium, families remain in Cartwright during the summer and thus have farther to travel to the bakeapple picking grounds (Fig. 3).

“I was a young teenager when the cod fishery was over so life began to change then. So life began to change and peoples’ way of life began to change from that day to this. Less and less and less people could make a livelihood from in shore fishing. Taking their families to summer fishing places.” Rosetta Howell

Few families can take a holiday to return to their summer homes, which can be hours away by boat, for bakeapple picking. More commonly, they will take day trips to nearby bakeapple picking grounds, less than a half hour away by boat.

“That’s the way we pick bakeapples. We run off for a few hours and go out for a day or two and come back.” Leslie Hamel

Community members explained that when not all the bakeapples are ripe they have to make multiple day trips back to the same berry patch over the course of the two weeks or so it takes all the bakeapples to ripen. It is more challenging for community members, in terms of finances and time, to deal with temporal and spatial variability in the ripening of bakeapples living farther from the bakeapple picking grounds.

Many families have increasing difficulty in finding the time to go bakeapple picking together. It is difficult to cope with variability in weather and harvest timing with rigid work schedules and increasingly, single men will go to the bakeapple grounds for a couple of hours.

### Adaptive Capacity

The adaptive capacity of the community informs how bakeapple pickers respond to the identified exposures and sensitivities. The speedboat, an upgrade from row-boats and then motor-boats, is a part of the community’s adaptive capacity because it enables families to travel more quickly and to continue frequenting their bakeapple picking grounds (Fig. 3).

“You jump on your boat and go on and you’re there in half the time you were there in a motor boat.” Rosetta Sainsbury

Because the speedboat reduces travel time, individuals can more easily get to a nearby bakeapple picking spot before or after work. The speedboat has -also encouraged families to explore new bakeapple picking spots.

“You can now go so much faster and so many different places.” Cookie Lethbridge In fact, some participants explain that the slower speed of motor-boats kept people from bakeapple picking at the best spots. Most families with a speedboat are better able to deal with the temporal and spatial variability of the bakeapple harvest.

Despite the advantages of the speedboat, challenges remain, including the difficulty of traveling long distances by boat, particularly for elders, high winds, the cost of gasoline, and access to a suitable boat.

“Getting ashore is always a problem if there is a sea on. Even if there is no wind sometimes you try and land and there’s too much sea. So you had to pick your days.” Leslie Hamel

Two participants considered the cost of gasoline to be a barrier to bakeapple picking. A woman, a picker for almost 70 years, said that she and her husband spend $40 per trip on gasoline making bakeapple picking too expensive compared to when she was growing up and could just walk to the bakeapple grounds. Also, two noted the difficulties arising from either not owning a boat or having rented out the boat for work.

The market for bakeapples is a part of the community’s adaptive capacity because it helps some individuals to offset the costs of gas. Individuals sell as much as 30 gal of bakeapples to supply the high demand in Happy Valley-Goose Bay (central Labrador) and on the island of Newfoundland where they are not abundant and as a result sell for ~$80 a gallon, as well as in Cartwright as a source of supplemental income. The market for bakeapples also caters to individuals in Cartwright who are unable to go out and pick bakeapples due to age, lack of resources, time, and/or interest (Fig. 3). In addition to the market for bakeapples, more than half of the interviewees said that they share their surplus bakeapples with family and friends, as well as others who are unable to pick for whatever reason.

“If I was going to get rid of any, I would give them away.” Donnie Howell

The TEK of generations of bakeapple pickers is a part of the community’s adaptive capacity because it captures when and where bakeapples are available and ripe. Pickers can start to predict the impact of weather on the ripening of bakeapples in the spring and communication among community members will identify the best harvesting grounds.

“We know not to go to certain places if we are looking for bakeapples because chances are slim you are going to get them… and a lot of that is local knowledge.” Judy Pardy

The value placed on bakeapples by individuals is also a part of the community’s adaptive capacity because it provides motivation to identify and adapt to ecological and social changes (Fig. 3). Almost all participants explained that the bakeapples are still a valuable part of the diet.

“Bakeapple picking has always been a big part of our lives for food. It’s been very important.” Donnie Howell

Also, interviewees prefer bakeapples to other fruits available at the grocery store and recognize their nutritional value.

“Whatever is in the grocery stores doesn’t matter. We are still going to get bakeapples if we can get them because they were something that we really liked to have, right?” (Anonymous male)

Participants also enjoy bakeapple picking because they get to be out on the land.

“For me, I love picking berries. I’d just go out and crawl around on the land all day long, you know?” Judy Pardy

Participants also pointed out that bakeapple picking is hard work and can be increasingly difficult with age. Some interviewees value the social aspect of bakeapple picking. After a long day of bakeapple picking, the family will meet up for a ‘boil up,’ which is a picnic with some hot tea. A third of participants continue to bakeapple pick because it is a tradition, but fewer brought up their Inuit ancestry as a reason for bakeapple picking since most largely associated bakeapple picking with their Labradorian culture.

“That’s how our ancestors survived and it just got passed down from generation to generation.” Dwane Burdett

A minority of participants observed that families, especially the younger generation, are less engaged in bakeapple picking.

“Like I said it is [bakeapple picking] getting done less and less. There are less and less people out on the land.” Dwane Burdett

“We still try and pick bakeapples every year. But even that families don’t do that anymore with their children or grandchildren. You know some do but very few.” Rosetta Howell

Because an increasing number of the younger generation places less value on family bakeapple picking and bakeapples as a food source, the cultural and nutritional value that families currently place on bakeapples may not be a source of adaptive capacity in the future.

## Discussion

The exposures, described by Cartwright community members, parallel observations in other arctic and subarctic communities. Bakeapple pickers explained that variability in the timing and abundance of bakeapples is to be expected. Some bakeapple pickers observed that the timing of picking has been earlier in the past decade largely due to earlier warming temperatures in the spring and summer (see also Downing and Cuerrier 2011). Some interviewees described how tree and larger shrub growth has encroached on bakeapple patches. First Nation communities in the Yukon and Northwest Territories, Inuit communities in Nunavik, and Gwich’in communities have also observed new shrub and tree species at berry picking spots (Parlee *et al.* 2005; Guyot *et al.* 2006; Cuerrier *et al.* 2015). Participants also observed that the berries ripen in a more fragmented way, are less abundant and smaller compared to when they were younger. A couple of participants attributed these changes to climate change. Climate change and vegetation growth have likely contributed to the decrease in palsas at The Big Marsh, consistent with trends seen in other permafrost-associated peatlands (Christensen *et al.* 2004; Johansson *et al.* 2006; Bouchard *et al.* 2014). The observed fragmentation of bakeapple patches may also be related to changing permafrost conditions in bogs. From field surveys showing that vegetation types are significantly different across palsa and no permafrost plots, and from satellite imagery revealing a decrease in palsas, we can infer that with continued palsa degradation, the vegetation will change and eventually resemble the present vegetation of the surrounding no permafrost areas. One bakeapple picker from Cartwright attributed the fragmentation of bakeapple patches and less abundant bakeapples to more extremes in the weather, including precipitation. Other Indigenous communities note changes in the bakeapples themselves. First Nation communities have described precipitation that is resulting in smaller berries and less snow bringing fewer berries (Guyot *et al.* 2006). In a Gitga’at community in British Colombia, residents explained that in recent years wild berries are scarce and attributed this to heavy rains (Turner and Clifton 2009).

In Cartwright, bakeapple picking is tied to other harvesting activities, specifically fishing. Similarly, in Gwich’in First Nation communities the harvest of berries and fish occur at the same time (Parlee *et al.* 2005). Other First Nation communities in British Colombia also harvest important plants, specifically edible seaweed, while fishing for salmon and halibut. As a result, any changes in the fishery have an impact on the harvesting of edible plants. In Cartwright resettlement impacted families’ land based livelihoods because it became more difficult for them to travel between their summer homes for fishing and berry picking and their winter homes. Similarly, resettlement programs also moved Inuit in Nunavut into permanent communities and ended their semi-nomadic way of living, which limited individuals’ ability to access hunting areas (Wenzel 2009) and reduced the extent of harvesting areas and the availability of country food (Ford and Beaumier 2011). Resettlement, with subsequent sociocultural transformations, is an example of a slow variable that has resulted in a change in culture and livelihoods over the past half century in Indigenous northern communities (Chapin *et al.* 2004; Ford *et al.* 2013).

New technology has enabled Indigenous communities to continue living off the land despite social and environmental change. In Cartwright, owning a speedboat allows families to continue visiting bakeapple picking grounds. Similarly, the snowmobile allows individuals in Inuit communities in Nunavut to continue hunting despite living farther away from traditional hunting grounds (Wenzel 2009). In Arctic Bay, Inuit use GPS and online sea ice reports to navigate changes in sea ice conditions (Archer *et al.* 2017). Members of a Gitga’at community in British Columbia created technology to dry their seaweed and fish despite increased precipitation (Turner and Clifton 2009).

The market for bakeapples in Cartwright is a part of the adaptive capacity of the community in that it provides supplemental incomes and makes it more affordable for many families to continue bakeapple picking. Community members did not indicate that the market for bakeapples has threatened traditional sharing networks with family and friends. Similarly, Inuit communities in Greenland have had widespread positive experiences selling country food for 150 years (Ford *et al.* 2016). In contrast, other Indigenous communities are concerned about the commercialization of traditional foods. For instance, in the Gwich’in settlement region, communities are concerned that commercializing wild berries will complicate traditional sharing networks (Murray *et al.* 2005; Parlee *et al.* 2005). Similarly, some Inuit communities in Nunavut have expressed concerns about the commoditization of country food in terms of traditional sharing networks (Ford *et al.* 2016; Searles 2016; see also MacDonald *et al.* 2013).

The cultural and nutritional value placed on bakeapple picking in Cartwright is also a key component of adaptive capacity. Other studies have documented the nutritional value that an Indigenous community in Alaska and an Inuit community in Nunavut also place on bakeapples (Downing and Cuerrier 2011; Kellogg *et al.* 2010). However, there is concern in Cartwright that younger families spend less time or no time bakeapple picking. Other studies document similar concerns within Indigenous communities that traditional knowledge is not being passed to younger generations (Turner and Turner 2008; Beaumier *et al.* 2015; Archer *et al.* 2017).

## Conclusion

Our results highlight social and physical changes that impact bakeapple picking from the perspective of the Indigenous community in Cartwright, Labrador. Interviewees described changes in temperatures, vegetation, and the consequent impacts on bakeapples. The impacts of these environmental changes on families’ abilities to harvest bakeapples are magnified by socioeconomic impacts, including the closures of the inland fisheries and the resettlement program of the 1960s. However, the advent of the speedboat, the commercial market for bakeapples, the cultural and nutritional value families continue to place on bakeapples, and concerns with maintaining traditional cultural and ecological knowledge seem to reinforce families’ continued engagement in bakeapple picking.

Contextual vulnerability to climate change encompasses not only the environmental impacts associated with climate change but the concurrent social, political, and economic processes that determine how a given community experiences climate change (Ford and Smit 2004; Smit and Wandel 2006; O’Brien *et al.* 2007; Bennett *et al.* 2016). Previous studies have described the contextual vulnerability of the land-based livelihoods of Indigenous communities in the Arctic (Ford *et al.* 2012a, b, 2015 Archer *et al.* 2017), and our study also illustrates that social and physical constraints to ease of access to traditional bakeapple picking grounds can impact harvesting capacity as much as the actual ecological impacts of climate change on the resource.
